# Spanish Adaptation and Validation of the Teaching and Learning Experiences Questionnaire

**DOI:** 10.3390/ijerph18073518

**Published:** 2021-03-29

**Authors:** María-Elena Parra-González, Jesús López-Belmonte, Adrián Segura-Robles, Antonio-José Moreno-Guerrero

**Affiliations:** 1 Department of Research Methods and Diagnosis in Education, University of Granada, 51001 Ceuta, Spain; elenaparra@ugr.es (M.-E.P.-G.); adrianseg@ugr.es (A.S.-R.); 2 Department of Didactics and School Organization, University of Granada, 51001 Ceuta, Spain; ajmoreno@ugr.es

**Keywords:** teaching environments, learning environments, validation, learning

## Abstract

Training processes are mainly based on the pedagogical methods applied by teachers. In many cases, these pedagogical methods are adapted to the social, economic, and cultural environment of the students themselves. In this study, we used a psychometric analysis based on the analysis of structural equations to detect the psychometric properties through classical goodness-of-fit indices. The objective of this study was to translate, adapt, and validate the instrument called the Teaching and Learning Experiences Questionnaire (ETLQ) for the population of Spanish adolescents in secondary education. The rrecommendations in the literature were followed for its translation and adaptation into Spanish. The results indicate that, after translation and adaptation, the model remained in 11 factors with acceptable goodness-of-fit indices. We conclude that the process of translation, adaptation, and validation of the ETLQ has produced a valid and reliable tool due to the psychometric findings revealed in the present work.

## 1. Introduction

Teaching and learning processes are subordinated to the pedagogical methods used by the teachers themselves, trying in many cases to adapt to the needs of the socio-economic and cultural environment of the students to whom the training is addressed [1]. The purpose of any pedagogical act is the learning of the student [2]. This fact, which, a priori, can be simple, has a set of implications for its proper development [3]. Among these aspects are the teaching and learning environments [4].

Learning environments can be considered as the different physical, contextual, and cultural situations in which the teaching and learning processes take place [1]. The environments that can be generated are diverse and broad [5], given that the pedagogical act can take place inside the classroom [6] or outside it [7], in either a natural environment [8] or a virtual one [9]. Furthermore, learning environments should not be focused solely on the physical environment [10]. The focus should extend to the cultural and contextual environment in which learning takes place [11]. These centre on pedagogical events that promote student learning [12]. That is, learning environments allow students to learn from anywhere [13], at any time [14], and in various ways [15].

There are several elements that must be taken into account in order to develop an appropriate learning environment [16]. These include physical [17] or virtual [18] environments; material and learning resources [19]; student characteristics [20]; the curricular elements to be developed during the teaching and learning process [21]; the activities to be developed [22]; the various evaluation strategies [23]; and the cultural process in which the pedagogical act takes place [24]. The teacher is partly responsible for all this and, therefore, is the one in charge of taking into account all these elements to adapt an adequate learning environment [25].

In order to carry out this type of analysis by the educational community, the Teaching and Learning Experiences Questionnaire (ETLQ) was developed [26] to identify aspects related to learning environments that encourage students to become involved in the pedagogical act [27,28]. Both the long version of the questionnaire and the short version of the questionnaire take into account, among other things, critical learning. This is divided into three dimensions: ability to think reasonably; ability to recognize alternative points of view; and ability to reflect on one’s own thoughts [29].

Other aspects analysed by the ETLQ questionnaire are the students’ approaches to learning and the evaluation of the pedagogical environment [30]. Among the students’ approaches to learning are the factors of determination, process, and product [31]. Under the determination factor is the commitment to learning [32]. This commitment is focused on two factors: personal, understood as the academic history of the students and their personal characteristics; and situational, focused on the organization of educational centres [33]. Among the process factors, the cognitive and metacognitive processes of the student are taken into account [34]. Finally, within the product factors, the results achieved by students in the teaching and learning process are measured [35]. In this case, hetero-evaluation, self-evaluation, and co-evaluation are all taken into account [36]. In these cases, hetero-evaluation is considered to be the teacher’s evaluation of the students, co-evaluation is the students’ evaluation of each other, and self-evaluation is the students’ evaluation of themselves.

It can be established, following current theory, that there are at least four fundamental elements for teaching and learning in the educational environment. These are the classroom context [37], the teaching and assessment of curricular elements [38], the relationship of students to their academic environment [39], and the culture of the learners [40]. In other words, these elements of the educational environment allow students to increase their commitment to learning and, therefore, to learn more effectively [41].

Various studies have shown how learning environments promote academic achievement [42]. In addition, they facilitate the development of skills in the subjects studied [43], that is, the acquisition of critical learning, improvements in communication, improvement in problem solving [44], and better interpersonal relationships among peers [45]. It can therefore be indicated that appropriate learning environments allow for adequate acquisition of the content and meaning of subject matter [46]. That is, there are connections between process factors and presage and product factors [47].

The ETLQ instrument has been adapted to different contexts, and there is a long version and a short version. The short version is the most widely used and suitable for various contexts [26]. This reduction has been produced in different adaptations, such as by reducing the number of scales [48], or the case of adaptation to the Finnish context, with a reduction in the number items [49], or adaptations and adjustments of the instrument with the intention of reducing its volume, in both number of items and number of dimensions [41,50]. In any case, the adaptations have been made in different contexts and at different times, with the intention of adapting the instrument to the cultural context.

The instrument [26], in its original English versions, contains 40 items analysing student perceptions of their learning environment—analysing aspects such as understanding, alignment, enthusiasm and personal support, interest and relevance, and constructive feedback and support from other students. It also has 18 items analysing approaches to study, oriented on scales of deep focus, surface focus, intention to understand, and organised study [26]. In the reduction in the manuscript, items measuring teaching and learning environments with eight dimensions were used, composed of a total of 25 items; these eight dimensions were aim and congruence, teaching for understanding, set work and feedback, assessment of understanding, enthusiasm and personal support, student support, interest, and choice [50]. The results of this study showed that, according to theory, the foreshadowing, process and product factors 3P learning model were associated with each other. In this case, it is considered that the psychometric properties of the instruments, in all versions, showed similar loadings and values [28].

## 2. Method

The objective of this study was to translate, adapt, and validate an instrument to a Spanish context [51,52]. The translation and adaptation procedures followed the guidelines described in the literature.

### 2.1. Instrument

The model and instrument to be adapted was a modified version of the Teaching and Learning Experiences Questionnaire (Appendix A). This scale was modified and validated in its original English version in [53]. The response options are of the Likert type (where 1 indicates “strongly disagree” and 5 indicates “strongly agree”). This model is composed of 11 factors, both first and second order. The first three factors evaluate teaching–learning, the learning environment for understanding,

The first three factors that evaluate the teaching–learning environment (teaching for understanding, disciplinary understanding, and supportive teaching) are indicators of a second-order factor called Fostering Learning. The remaining three environment factors are alignment, peer support, and constructive feedback. Three factors (deep focus, shallow focus, and organized study) assess students’ learning approaches. In addition, elements related to critical thinking were added to the questionnaire.

### 2.2. Survey Translation

To translate questionnaires, there are no fixed methodological guidelines to be followed [54], as they will also require cultural adaptations in most cases [55]. However, there are recommendations, such as those proposed by [56], to translate scales or models as efficiently and systematically as possible. The guidelines that should be followed and that have been followed in this case are these moments:

- Moment 1. Two bilingual experts review the English document and analyse whether or not it is feasible to translate the questionnaire.
- Moment 2. The authors and/or researchers translate the questionnaire into Spanish.
- Moment 3. The above-mentioned experts and two monolingual researchers review the translation and make the necessary modifications in each case.
- Moment 4. The bilingual experts translate the scale or questionnaire into Spanish, and then it is checked for concordance and coherence with the translations of the same authors and/or researchers, thus guaranteeing that the scale or questionnaire is as faithful as possible to the original English version.

### 2.3. Data Collection

Data collection was carried out in Ceuta (Spain). It occurred during the time of the pandemic, specifically between April and May 2020. The data were collected using our own platform for information collection (Limesurvey Services), which allowed us to both conduct the analyses and prepare the data matrix for subsequent analyses. Accidental sampling was used as it allows the researchers to access the participants in a faster and more efficient way. Two educational centres from the aforementioned context participated. In addition, several groups of the secondary education stage were selected. The translated survey used for data collection is given in Appendix A. Once all the data were collected, they were downloaded in matrix form. The next step was to enter them into SPSS statistical analysis software version 25 for further analysis. Regarding gender, the final sample was composed of 56.4% male and 43.6% female participants with an age range of 11 to 16 years with a mean age of 14.38. Final sample of 307 secondary students was recruited.

### 2.4. Data Analysis

The data analysis was carried out in two phases. In the first phase, all the constructs proposed in the model of the modified version of the scale by Utriainen et al. [28] were tested, with structural analyses carried out to verify the theoretical model proposed by the original authors [26]. In the second phase, after checking that the model fitted in a detailed way and that there were no major structural or theoretical problems, a validation of the complete model and all the proposed relationships was carried out. A system of structural equations was also used to obtain the indices of goodness of fit for the model in its entirety. Factorial analyses were used to check the structure of an instrument with respect to a theoretical construct are complemented with the use of classic goodness-of-fit indices as comparative fit index (CFI), Tucker–Lewis index (TLI), Chi-squared (CMIN), Standarized Root Mean-Square (SRMS) and other reliability indicators such as the Cronbach’s Alpha [27].

## 3. Results

**Figure 1:**
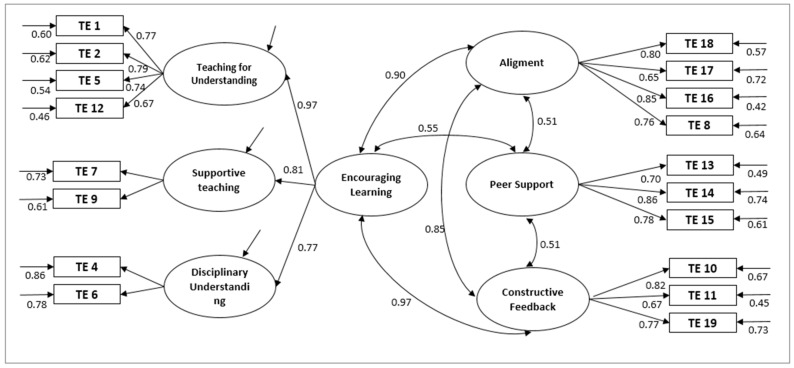
Original model for items and dimensions pertaining to students’ perceptions of the learning environment (**a**).

At first, the original English model was tested. As it is a complex model, the structural validations were performed first by separating the main dimensions. Specifically, for the dimension “Teaching–Learning Environment” a second-order factorial model was proposed, which, in turn, was made up of six dimensions: “Teaching for understanding”, “Supportive Learning”, “Disciplinary understanding”, “Peer Support”, “Alignment”, and “Constructive feedback” (Figure 1). As with the authors’ original proposal, problems were found with the correlations of Item 3 (“I enjoy this way of learning”), so it was decided to follow the original model proposed by the authors.

**Figure 2:**
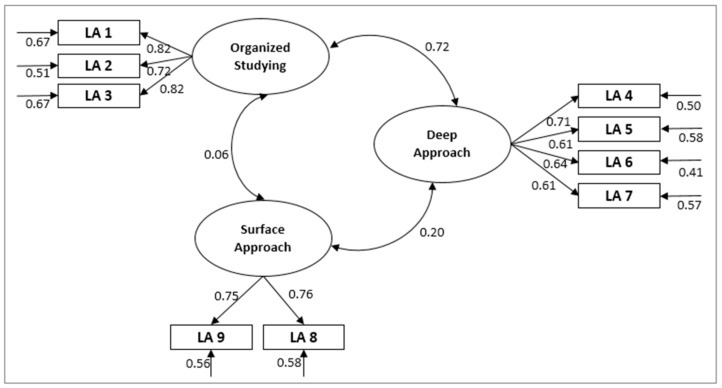
Measurement model for student approaches to learning factors (**b**).

In the same way, in Figure 2, the adjustment indices for the student approaches to learning factors proposed by the original authors are presented. For the model on students’ perceptions of the learning environment (A), acceptable values were obtained that indicated a good fit of the model (CFI = 0.926; TLI = 0.936). All estimated parameters were statistically significant at *p* < 0.001. The modified measurement model for student approaches to learning factors (B) was then tested. Adjustments for the construct could also be considered good (CFI = 0.969; TLI = 0.968) with *p* < 0.001. In this case, unlike in the original model, the correlations found for Surface Approach–Deep Approach and Surface Approach–Organized Studying were not negative (r = 0.06, r = 0.20, respectively), but they were much weaker than that found for Organized Studying–Deep Approach (r = 0.72).

**Figure 3:**
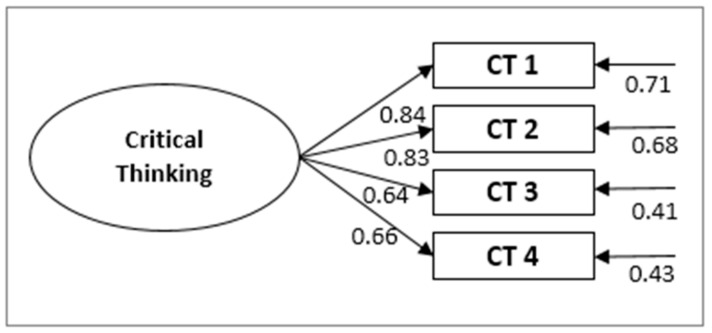
Measurement model for critical thinking (**c**).

Then, the critical thinking model was tested, and all the values obtained in the original model and in the one translated to the Spanish context seem to show good adjustment indices (CFI = 0.946; TLI = 0.947) with the parameters obtained being statistically significant at *p* = 0.018. In this case, Item 3 (“I have learned to apply the theoretical knowledge to practice”) and Item 4 (“I have learned to develop new ideas”) seemed to be highly related, so we chose to eliminate Item 4 (Figure 3).

**Figure 4:**
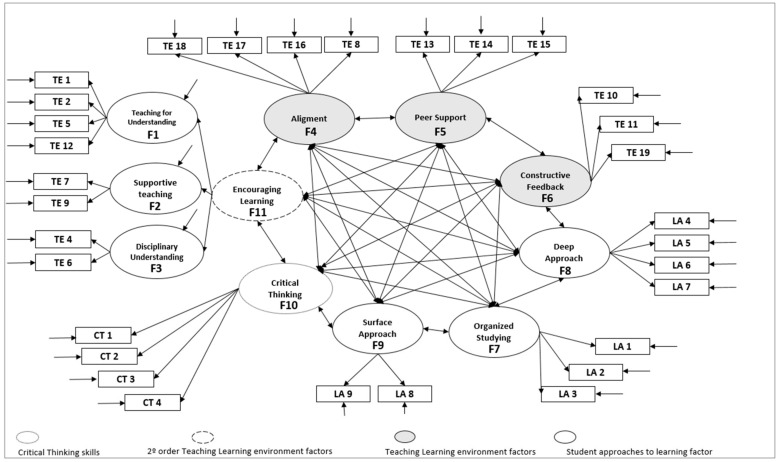
Proposed original model with all dimensions included.

Likewise, the complete model was tested; the relationships of all the dimensions proposed by the authors were analysed to check their structure after the translation and adaptation was carried out (Figure 4). The adjustment indices obtained were still correct, except for the value χ^2^(404) = 801.4, *p* < 0.001, CMIN= 1.999. The values obtained for the different indices were CFI = 0.912, TLI= 0.90, SRMR= 0.057, and RMSEA = 0.05 (90% CI = 0.51, 0.63), which can be considered acceptable or excellent. It should be noted that the CFI and TLI indices were significantly below the 0.95 cut-off point indicating an excellent model, results which coincide with those for the original model (Table 1).

The factor loads and alpha indices are presented in full in Table 2 and Figure 4. Most of the estimators for the complete model were significant at *p* < 0.001, except for the correlation between the factors F7 (Deep Approach) and F9 (Surface Approach) with a *p*-value of 0.008. When comparing the results obtained from translating and testing the model with those from the original model in English, similar values were found for the “teaching–learning environment” dimension; however, the values of 0.84 for TE9 with respect to 0.67 in the original version, 0.81 for TE18 with respect to 0.70, and 0.63 for TE13 with respect to 0.75 stand out. In the same way, for the items that make up the “student approach to learning”, we found values of 0.80 for LA3 with respect to 0.60, and 0.75 for LA8 with respect to 0.55. For the section “Critical Thinking”, only CT1 stood out, with 0.81 compared to 0.67 originally.

Finally, Table 3 shows the correlations between all the factors proposed in the original model. As can be seen, almost all the factor relations were significant, except for the relations established by F9 (“Surface Approach”) for F4, F5, F6, and F7. Furthermore, unlike in the original model, none of the significant relationships found were negative, with all of them being positively related.

## 4. Discussion

This study was developed with the aim of adapting and validating the ETLQ instrument for the Spanish context, specifically for the adolescent population. Achieving this objective was possible, as shown in the results, due to the various statistical procedures carried out that led to a modification of the model by means of confirmatory factor analyses, certifying the relevance and validity of the designed tool.

There is no doubt about the wide spectrum of learning environments that can currently be generated [1,5]. The impact of technology in the educational field has allowed the creation of various learning spaces [15]. The new learning environments are no longer only anchored to a specific physical classroom—learning can be exported anywhere [6,7]. This allows students and teachers to break the space and time barriers that have traditionally characterized the formative process [13]. Thus, technology has given a ubiquitous component to the teaching and learning process [14].

In order to know the suitability and relevance of the learning environment generated and experienced, it is essential to have adequate instruments that are validated and, especially, contextualized to the peculiarities of the region of the population under study [56]. All this is necessary to collect accurate data adjusted to the reality being analysed [57]. In this sense, the adaptation of previously designed tools for certain contexts is a necessary field of research to give depth to the scientific literature. In this line of research, it is possible to increase the repertoire of validated instruments to measure a certain construct, taking into account the singularities that characterize each geographical context. Likewise, these works have an impact on decreasing the biases derived from the use of inappropriate tools, as well as allowing for the achievement of reliable results [58].

The ETLQ has been adapted to different regional settings. This has promoted the appearance of various versions, with the number of dimensions and, consequently, the number of items varying among them [26]. Reduction with respect to the original has been carried out in different studies, where the scales [48] and number of items [49] were reduced, and even substantial modifications were made in the number of items and dimensions that articulate them [41,50].

In each of the contextual adaptations and subsequent validations of the tool, statistical values were reached that ensured the suitability of the instrument in such contexts. This study has obtained adequate and relevant psychometric properties with respect to its predecessor validations. Therefore, this instrument is positioned as an optimal and improved tool with respect to previous versions of other contexts [26,41,48,49,50]. With all the above in mind, in this study, the ETLQ was adapted and validated to the Spanish context in order to allow for analysis of the online learning experiences that have emerged as a consequence of the transformation that coronavirus disease 2019 (COVID-19) has caused in learning spaces [59], going from a purely face-to-face and sometimes hybrid scenario to distance learning [60].

## 5. Conclusions

Particularly, in this study, given the statistical analysis carried out, it is concluded that the translation of the ETLQ, subsequently adapted and validated to the Spanish context for the adolescent population, is a valid and reliable instrument. Therefore, this instrument is an ideal tool to carry out measurements on the different learning environments that can be developed today to carry out the process of transmission and generation of knowledge among teachers in the Spanish learning context and students in secondary education.

The present work found limitations in its development. As a consequence of the pandemic caused by COVID-19, the availability of the sample was affected. This caused a delay in the investigation because many participants did not have the necessary resources to fill in the questionnaire digitally. In response to this situation, the school in question provided electronic devices to the students most affected by the digital divide. This action finally allowed the collection of data of an adequate sample size to validate the instrument.

As a future line of study, the intention is to apply this instrument in different territories of Spain. All this is with the intention of checking the effectiveness of the learning environments used in Spain during the COVID-19 pandemic and, thus, to be able to make a generalization of these findings to the whole population. Furthermore, we propose the development of an instrument that addresses, in addition to the questions raised in this study, questions about the quality or adequacy of a course or for the evaluation of a teacher.

This study has several implications that reveal the potential of this instrument today. With respect to the theoretical implications, with the realization of this investigation, a valid tool has been made to analyse the learning environments of Spanish students in secondary education. As far as practical implications are concerned, this work offers different professionals in education a tool to analyse the learning environments, as well as to verify the potentialities and weaknesses of each of them. At the present time, an increase in the use of digital learning environments has been projected in Spain as a consequence of COVID-19. Therefore, it is necessary to have an appropriate instrument to assess these training practices. With the study of these data, improvement proposals and teacher training plans can be established to increase the quality and improve the design of these environments. Likewise, this study can serve as a basis for the adaptation of the instrument to other educational stages.

## Figures and Tables

**Table 1:** Goodness-of-fit indices for the models analysed.

	χ^2^	df	*p*	RMSEA	RMSEA 90% CI	CFI	TLI
(a) Model on student perceptions of the learning environment.	3143	128	0.000	0.06	0.059–0.079	0.926	0.931
(b) Measurement model for student approaches to learning factors	43,797	25	0.011	0.05	0.023–0.073	0.969	0.968
(c) Model on critical thinking	5514	2	0.018	0.03	0.024–0.040	0.946	0.947

**Table 2:** Standardized load factors and alpha values for the complete model adapted and analysed.

	First-Order Factors										Second-Order Factor
Item	F1	F2	F3	F4	F5	F6	F7	F8	F9	F10	F11
TE1	0.62										
TE2	0.71										
TE5	0.75										
TE12	0.65										
TE7		0.75									
TE9		0.84									
TE4			0.63								
TE6			0.74								
TE16				0.76							
TE17				0.63							
TE18				0.81							
TE8				0.73							
TE13					0.68						
TE14					0.84						
TE15					0.77						
TE10						0.78					
TE11						0.63					
TE19						0.34					
LA1							0.80				
LA2							0.71				
LA3							0.80				
LA4								0.71			
LA5								0.63			
LA6								0.61			
LA7								0.60			
LA8									0.75		
LA9									0.76		
CT1										0.81	
CT2										0.77	
CT3										0.71	
CT4										0.73	
F1											0.73
F2											0.78
F3											0.98
Cronbach	0.781	0.772	0.645	0.824	0.808	0.785	0.823	0.735	0.726	0.844	----

**Table 3:** Correlations between factors in the Teaching and Learning Experiences Questionnaire (ETLQ) model by Utriainen et al. (2018) [29].

Factor	F4	F5	F6	F7	F8	F9	F10	F11
F4 Alignment	--							
F5 Peer Support	0.35 ***	--						
F6 Constructive Feedback	0.25 ***	0.17 ***	--					
F7 Organized Studying	0.46***	0.30 ***	0.20 ***	--				
F8 Deep Approach	0.40 ***	0.31 ***	0.20 ***	0.48 ***	--			
F9 Surface Approach	0.033	0.90	0.06	0.53	0.15 **	--		
F10 Critical Thinking	0.42 ***	0.32 ***	0.23 ***	0.50 ***	0.41 ***	0.21	--	
F11 Encouraging Learning	0.60 ***	0.36 ***	0.31 ***	0.45 ***	0.46 ***	0.11	0.50 ***	--

** *p* < 0.05 *** *p* < 0.001.

## Data Availability

Data are contained within the article.

## Appendix A

**Entorno de Enseñanza y Aprendizaje (TE)**	
TE1	Los contenidos en general, fomentan que pueda relacionar lo que he aprendido con el mundo real
TE2	Puedo ver la importancia de la mayoría de lo que nos enseñan
TE3	Disfruto de esta forma de aprender
TE4	El profesorado nos ayuda a aprender cómo pensar y llegar a conclusiones sobre las cosas
TE5	Esta forma de enseñarnos me ayuda a pensar en las pruebas que sustentan los diferentes puntos de vista
TE6	Esta forma de enseñarnos y aprender me ha dado una idea de lo que pasa “entre bastidores”
TE7	El profesorado trata de compartir su entusiasmo con nosotros
TE8	Lo que nos enseñan parece coincidir con lo que se supone que debíamos aprender
TE9	El profesorado es paciente al explicar cosas que parecen difíciles de entender
TE10	La retroalimentación o respuesta que se da a mi trabajo me ayuda a mejorar mi forma de aprender y estudiar
TE11	Los comentarios sobre mi trabajo me ayudan a aclarar cosas que no había entendido del todo
TE12	Las tareas me ayudan a relacionarlas con mi conocimiento o experiencia previa
TE13	Puedo trabajar cómodamente con otros compañeros y compañeras
TE14	Entre compañeros nos apoyamos y ayudamos cuando es necesario
TE15	Hablar con otros compañeros me ayuda a desarrollar mi comprensión
TE16	Tengo claro lo que se espera de las tareas para la evaluación
TE17	Puedo ver cómo las tareas encajan con lo que se supone que debemos aprender
TE18	Tengo claro lo que se supone que tengo que aprender
TE19	Recibo normalmente comentarios de los profesores sobre mis tareas
**Enfoque del aprendizaje del estudiante (LA)**	
LA1	En general, soy bastante sistemático y organizado en mis estudios
LA2	Generalmente me esfuerzo en mis estudios
LA3	He organizado mi tiempo de estudio cuidadosamente para hacer el mejor uso de él
LA4	Miro con cuidado la evidencia antes de llegar a mi propia conclusión sobre lo que estoy estudiando
LA5	Las ideas que encuentro en mis lecturas académicas a menudo me llevan a largas cadenas de pensamiento
LA6	Cuando comunico ideas, pienso en lo bien que transmito mis puntos
LA7	Si no entiendo bien las cosas al estudiar, intento un enfoque diferente
LA8	A menudo tengo problemas para encontrarle sentido a las cosas que tengo que recordar
LA9	Mucho de lo que aprendo parece un montón de piezas sin relación en mi mente
**Pensamiento Crítico (CT)**	
CT1	He aprendido a analizar y organizar la información
CT2	He aprendido a evaluar los temas de forma crítica
CT3	He aprendido a aplicar los conocimientos teóricos a la práctica
CT4	He aprendido a desarrollar nuevas ideas
